# Relevance of interferon-gamma in pathogenesis of life-threatening rapidly progressive interstitial lung disease in patients with dermatomyositis

**DOI:** 10.1186/s13075-018-1737-2

**Published:** 2018-10-26

**Authors:** Yuichi Ishikawa, Shigeru Iwata, Kentaro Hanami, Aya Nawata, Mingzeng Zhang, Kaoru Yamagata, Shintaro Hirata, Kei Sakata, Yasuyuki Todoroki, Kazuhisa Nakano, Shingo Nakayamada, Minoru Satoh, Yoshiya Tanaka

**Affiliations:** 1 The First Department of Internal Medicine, School of Medicine, University of Occupational and Environmental Health, Japan, 1-1 Iseigaoka, Yahatanishi-ku, Kitakyushu City, 807-8555 Japan; 2 Department of Pathology and Cell Biology, School of Medicine, University of Occupational and Environmental Health, Japan, Kitakyushu City, Japan; 3 Department of Clinical Immunology and Rheumatology, Hiroshima University Hospital, Hiroshima, Japan; 4 Mitsubishi Tanabe Pharma Corporation, Yokohama, Japan; 5 Department of Clinical Nursing, School of Health Sciences, University of Occupational and Environmental Health, Japan, Kitakyushu City, Japan

**Keywords:** Rapidly progressive interstitial lung disease, Dermatomyositis, IFN-γ

## Abstract

**Background:**

Dermatomyositis (DM) with rapidly progressive interstitial lung disease (DM RP-ILD) is a life-threatening condition. Serum cytokine levels are potentially suitable biomarkers for DM RP-ILD. However, the relationships among cytokine levels, lung imaging findings, and lung pathology have not been investigated. The aim of the present retrospective study was to determine the association between hypercytokinemia and lung inflammation in patients with DM RP-ILD.

**Methods:**

The study subjects were nine patients with life-threatening DM RP-ILD and severe hypoxemia (partial arterial oxygen pressure (PaO_2_)/fraction of inspired oxygen (FiO_2_) ratio ≤ 200) before receiving intensive care management, who were admitted to our hospital between 2006 and 2015. The controls included 10 patients with DM without RP-ILD and 19 healthy subjects. We assessed the association between serum cytokine levels and computed tomography (CT) scores of the lung (ground glass opacity-score, G-score; fibrosis-score, F-score). Lung, hilar lymph nodes, and spleen from two autopsies were examined by hematoxylin-eosin (H&E) staining and immunostaining.

**Results:**

Serum interferon (IFN)-γ, interleukin (IL)-1β and IL-12 levels were significantly higher in patients with DM RP-ILD than in the other two groups, whereas serum IL-6 levels were elevated in the two patient groups but not in the healthy subjects. Serum levels of IL-2, IL-4, IL-8, IL-10, IFN-α, and TNF (tumor necrosis factor)-α were not characteristically elevated in the DM RP-ILD group. Serum IFN-γ levels correlated with G-scores in patients with DM RP-ILD, while IL-1β was negatively correlation with F-scores. Immunohistochemical staining showed infiltration of numerous IFN-γ-positive histiocytes in the lung and hilar lymph nodes; but not in the spleen. Serum IL-6 levels did not correlate with the CT scores. Numerous IL-6-positive plasma cells were found in hilar lymph nodes, but not in the lungs or spleen.

**Conclusions:**

Our results suggest strong IFN-γ-related immune reaction in the lungs and hilar lymph nodes of patients with life-threatening DM RP-ILD, and potential IFN-γ involvement in the pathogenesis of DM, specifically in the pulmonary lesions of RP-ILD.

**Electronic supplementary material:**

The online version of this article (10.1186/s13075-018-1737-2) contains supplementary material, which is available to authorized users.

## Background

The rate of interstitial lung disease (ILD) in patients with dermatomyositis (DM) is approximately 30% [1, 2]. While most patients exhibit slow progression of ILD, some exhibit rapidly progressive ILD (RP-ILD), in which the respiratory status deteriorates rapidly within 2–3 months from the onset of ILD [3–5]. In particular, a high incidence of RP-ILD has been reported in patients with clinically amyopathic dermatomyositis (cADM) who are positive for anti-melanoma differentiation-associated gene 5 (MDA5) antibodies (Abs) [6, 7]. RP-ILD in cADM is extremely difficult to treat and associated with a high mortality rate. Kameda et al. [8] reported the efficacy of intensive therapy with high-dose glucocorticoids (GC), intravenous cyclophosphamide (IVCY), and cyclosporine-A (CsA) in patients with DM complicated with RP-ILD (DM RP-ILD). Nakashima et al. [9] also reported marked improvement in prognosis of anti-MDA5 Abs-positive patients with DM using the same regimen, from the early stages of RP-ILD, with 75% survival rate by intensive immunosuppressive regimen versus only about 29% by conventional step-up therapy. Despite these encouraging reports, poor prognosis has been reported even in patients on intensive therapy, such as those with anti-MDA5 Abs-positive cADM, with a mortality rate after 6 months of treatment of as high as 25% [8]. In a retrospective analysis of 56 patients (including 49 patients with RP-ILD) treated in the intensive care unit (ICU) for exacerbation of DM/polymyositis (PM), Peng et al. [10] reported an overall survival rate of 14% (*n* = 8 out of 56), though the survival rate after 28 days was 0% in patients with cADM. Thus, the prognosis of anti-MDA5 Abs-positive cADM patients with RP-ILD is poor, as is the prognosis of patients with DM who develop RP-ILD during the course of treatment. Although it has been reported that treatment with tacrolimus (TAC), a calcineurin inhibitor, similar to CsA, and rituximab (RTX), is effective for life-threatening DM RP-ILD refractory to the above intensive therapy [11–13], this outcome remains to be confirmed.

Almost all anti-MDA5 Abs-positive patients have cADM with a high incidence of acute or subacute ILD [6, 14]. In a retrospective analysis of 13 patients with anti-MDA5 Abs-positive cADM, Takada et al. [15] reported that mortality was associated with high levels of anti-MDA5 Abs, suggesting that the levels of anti-MDA5 Abs could be useful in predicting prognosis. Since a strong association between DM RP-ILD and anti-MDA5 Abs has been confirmed previously in several studies, research on the pathophysiology of DM RP-ILD has been conducted mainly in anti-MDA5 Abs-positive patients [16]. High serum levels of ferritin and several types of inflammatory cytokines have been described in patients with DM RP-ILD [17–21], suggesting their involvement in the pathogenesis of RP-ILD. The pathophysiology of DM RP-ILD could be similar to that of macrophage activation syndrome (MAS), in which a variety of cytokines (e.g., interleukin (IL)-1, IL-6, tumor necrosis factor (TNF)-α) are involved [22]. However, despite studies suggesting that serum cytokines levels could be useful biomarkers for monitoring disease activity and to predict the prognosis of DM RP-ILD, the associations among serum cytokine levels, pulmonary image findings (e.g., computed tomography (CT) score) and lung pathology, have not been investigated thoroughly. The present study was designed to determine the relationships among serum cytokine levels, CT scores of the lung, and the histopathologic assessment of lung tissue.

## Methods

### Study design and patients

This study included nine Japanese patients with DM, aged ≥ 20 years, who had life-threatening RP-ILD and were admitted to our department between 2006 and 2015 and treated at the in-patient intensive care management unit. The term RP-ILD is not well-established and is used mainly by rheumatologists but not by pneumologists. Since we understand that the lack of standardization of the term RP-ILD can cause clinical bias and confusion, we defined RP-ILD with reference to the definition of acute respiratory distress syndrome (ARDS) in this study [23, 24]. Life-threatening RP-ILD was defined based on previous reports [7, 23, 24] as “a critical condition characterized by severe hypoxemia (PaO_2_/FiO_2_ ratio ≤200) that progressed within 3 months before initiation of treatment or intensification”. The control groups included 10 patients with DM with ILD (that did not meet the definition of RP-ILD) who underwent high-dose GC therapy (equivalent to prednisolone (PSL) of > 1 mg/kg/day) and 19 healthy individuals. Age-matched patients with DM without RP-ILD were randomly selected from the cohort of patients with DM/PM who were admitted to our department (*n* = 38) between 2014 and 2015. Thus, the total number of subjects in this study was 38. With regard to evaluation of serum cytokines, the major cytokines (IL-1β, IL-2, IL-4, IL-6, IL-8, IL-10, IL-12, interferon (IFN)-γ, IFN-α, TNF-α) involved in DM RP-ILD and MAS were selected based on the literature [17–22]. Cytokine levels were measured in all disease groups before the initiation or intensification of the treatment.

For patients with DM RP-ILD, the CT scores of the lung (ground glass opacity (GGO) score (G-score), fibrosis score (F-score)) and their association with serum cytokine levels were analyzed. This study was approved by the institutional review board of our university (#H28–033).

#### Diagnostic criteria

The diagnosis of DM was based on the Bohan and Peter criteria for PM/DM while that of cADM was based on the diagnostic criteria of Euwer and Sontheimer [25–28].

#### Exclusion criteria

Patients with pulmonary lesions due to bacterial pneumonia, fungal pneumonia, or pneumocystis pneumonia (PCP) and those with sepsis were excluded. Bacterial pneumonia was diagnosed based on positive sputum culture and detection of bacteria phagocytosed by leukocytes. Fungal pneumonia was diagnosed based on positive sputum or bronchoalveolar lavage fluid (BALF) culture for fungi, high serum β-D-glucan levels, positivity for antigens of Candida, Aspergillus or Cryptococcus, and chest CT findings consistent with fungal pneumonia. PCP was diagnosed based on positive polymerase chain reaction (PCR) of the sputum or BALF for *Pneumocystis jirovecii*, and chest CT findings consistent with PCP. Sepsis was diagnosed based on The Third International Consensus Definitions for Sepsis and Septic Shock [29].

#### RP-ILD assessment by CT scores

**Fig. 1:**
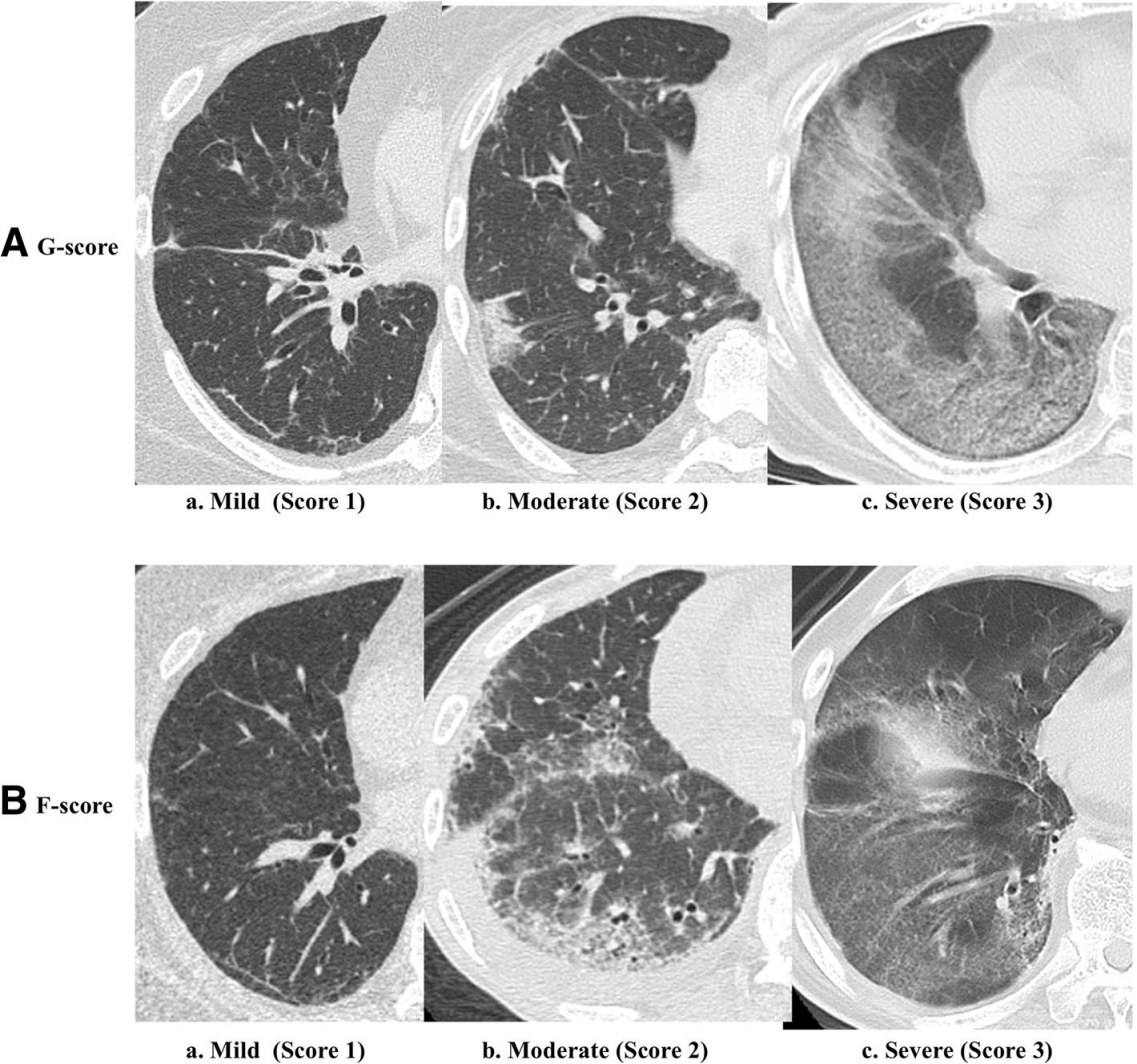
Assessment of rapidly progressive interstitial lung disease (RP-ILD) by computed tomography (CT) scores. **A** Assessment of RP-ILD by CT ground glass opacity (GGO) scores (G-scores): (**a**) thin-section CT scan shows small areas with GGO compared with normal parenchyma at the right lower lobe (mild GGO = 1); (**b**) CT scan shows extensive GGO that could be easily identified when compared with the normal parenchyma at the right lower lobe (moderate GGO = 2); (**c**) thin-section CT scan shows areas with diffuse GGO at the right lower lobe (severe GGO = 3). **B** Assessment of RP-ILD by CT fibrosis scores (F-scores): (**a**) thin-section CT scan shows areas with thickened interlobular septum or predominant peripheral fibrosis (mild fibrosis = 1). (**b**) CT scan shows extensive fibrosis that could be easily identified when compared with normal parenchyma at the right lower lobe (moderate fibrosis = 2), moderate fibrosis and bronchiolectasis. (**c**) thin-section CT scan shows areas with diffuse fibrosis at the right lower lobe (severe fibrosis = 3). Note honeycombing, bronchiectasis, peribronchovascular thickening, and subpleural cysts

Lung CT was evaluated semi-quantitatively using two-types of CT score; the G-score, which reflects changes in the acute and active phases, and the F-score, which reflects changes mainly in the chronic phase. Images were scored by two rheumatologists with at least 15 years of clinical experience, who were blinded to the demographic and clinical information. The left and right lung fields were divided into three regions and total of six lung zones were scored separately: upper (aortic arch zone), middle (tracheal bifurcation zone), and lower (supradiaphragmatic zone). The G-score and F-score of each zone were scored on a scale of 0–3 (maximum score = 3 points). The final CT score used for the analysis was the mean score of the six zones assessed by the two rheumatologists. The criteria used for the G-score were as follows: 1 point for predominantly subpleural partial GGO (Fig. 1A-a), 2 points for more pronounced GGO relative to that of G-score 1 point (Fig. 1A-b), and 3 points for diffuse GGO extending over a wide area (Fig. 1A-c). On the other hand, the criteria used for the F-score were as follows: 1 point for thickening and fibrosis of parts of the interlobular septa, mainly in the subpleural area (Fig. 1B-a), 2 points for more pronounced fibrosis and bronchiectasis compared with that for 1 point (Fig. 1B-b), and 3 points for diffuse and widespread fibrosis, honeycomb lung, and bronchiectasis (Fig. 1B-c) [30–32].

#### Endpoints and clinical assessment

The primary endpoint was elucidation of the significance of the elevated cytokines in DM RP-ILD. The secondary endpoint was the correlation between serum cytokines and CT scores in DM RP-ILD.

#### Measurements of serum cytokine levels

We measured the serum concentrations of various cytokines (IL-1β, IL-2, IL-4, IL-6, IL-8, IL-10, IL-12, IFN-α, IFN-γ, TNF-α) at the time of admission. Serum samples were isolated and stored at − 80 °C until analysis. The concentrations of these cytokines were measured by cytometric bead array (Becton Dickinson, Franklin Lakes, NJ, USA) using a FACSVerse flow cytometer (Becton Dickinson). Data were analyzed using the FCAP Array software (Becton Dickinson).

#### Immunohistochemical analysis

Immunohistochemical analysis was performed as described previously [33]. Antigen retrieval was performed by soaking the specimen on slides in 5 M sodium citrate solution in phosphate-buffered saline containing 0.05% (*v*/v) Tween 20 (PBST) (pH 6.0). The slides were subsequently blocked with serum-free protein block (Dako, 2016–08) for 30 min at room temperature and incubated at 4 °C overnight optimally with rabbit polyclonal antibodies to IFN-γ (ab25101, Abcam Inc., Boston, MA, USA) or IL-6 (21865–1-AP, Proteintech Inc., Rosemont, IL, USA) diluted 1:200 in DAKO antibody diluent. After washing three times with PBST, the slides were incubated with anti-rabbit IgG secondary antibody conjugated with horseradish peroxidase-labeled polymer (DakoCytomation, Glostrup, Denmark) and subsequently visualized by treatment with 3,3′ diaminobenzidine (DAB) Chromogen (DakoCytomation, #K3465) according to the instructions provided by the manufacturer. Nuclei were visualized using Mayer’s hematoxylin (MERCK, 1:1000 dilution in PBST). For mounting, the sections were rinsed with water, dehydrated in graded ethanol (90% ethanol for 30 s × 3 and 100% ethanol for 30 s × 3), cleared in xylene (for 30 s × 2), and sealed using multi-mount 480 (Matsunami, FM48001). Images were acquired and processed digitally.

#### Measurement of myositis-specific autoantibodies (MSAs)

We tested all serum samples by immunoprecipitation and enzyme-linked immunosorbent assay (ELISA) using recombinant proteins for anti-MDA5, anti-Jo-1, centromere protein A (CENP-A), CENP-B, Ro-52, and Ro-60 Abs. In addition, in patients positive for anti-aminoacyl-transfer ribonucleic acid synthetase (anti-ARS) Abs, the levels of anti-glycyl-tRNA synthetase (anti-EJ), anti-threonyl-tRNA synthetase (PL-7), anti-PL12, and anti-KS Abs were analyzed by ELISA.

#### Immunoprecipitation

Myositis-specific autoantibodies in serum were analyzed by immunoprecipitation of K562 cell extracts radiolabeled with ^35^S-methionine as described previously [34]; the specificities of the autoantibodies were determined using specific reference serum. Analysis of RNA components of the immunoprecipitates was also performed when necessary.

#### Anti-MDA5 and Jo-1 ELISA

Anti-MDA5 and Jo-1 Abs were tested by enzyme-linked immunosorbent assay, using recombinant proteins (0.5 μg/ml; Diarect AG, Freiburg, Germany) and 1:250 diluted serum, as described previously [34, 35]. The optical density was measured and converted into units using a standard curve created by a prototype-positive serum. The specificity of ELISA-positive serum was confirmed by immunoprecipitation.

#### Statistical analysis

Continuous variables were reported as mean plus or minus standard deviation or median (interquartile range). Differences between two groups were compared using the Mann-Whitney U test. Differences among multiple groups were compared using the Kruskal-Wallis test, followed by post-hoc Dunn’s multiple comparison test. Multiple group tests using the median test were also performed to determine the median values; the Wilcoxon test was used as the post-hoc test. The correlation between serum cytokine levels and CT scores was calculated using Spearman’s correlation coefficient. Statistical significance was set at *p* < 0.05. Statistical analyses were performed using the JMP version 9.0 (SAS Institute Inc., Cary, NC, USA).

## Results

### Demographic data of patients

The demographic data of patients in the DM RP-ILD groups are summarized in Table 1. The disease duration of DM with and without RP-ILD was 18.1 ± 39.8 and 7.6 ± 8.7 months, respectively (Table 1). Among the 19 patients, 11 were considered to have new-onset untreated anti-MDA5 Abs-positive DM. All six anti-MDA5 antibody-positive patients with DM with RP-ILD had hypoxemia (partial arterial pressure of oxygen (PaO_2_)/fraction of inspired oxygen (FiO_2_) ratio ≤ 200) before starting intensive therapy and their disease duration was 1.2 ± 0.4 months. On the other hand, none of the five anti-MDA5 antibody-positive patients with DM who were free of RP-ILD were hypoxemic before the start of treatment, and their disease duration was 5.5 ± 4.3 months.

**Table 1:** Clinical characteristics of patients

	DM with RP-ILD	DM without RP-ILD	*p* value
n	9	10	
Age, years	69.3 ± 3.9	63.9 ± 14.2	0.68
Female (*n*, %)	8, 88.9	6, 60.0	0.31
Disease duration (months)	18.1 ± 39.8	7.6 ± 8.7	0.40
Smokers (current and past) (%)	11.1	30.0	0.31
Number of GC pulses	2.2 ± 1.1	N/A	
PaO_2_/FiO_2_ ratio	160 ± 90	N/A	
Leukocyte count (/μL)	9438 ± 5751	7620 ± 5458	0.35
LDH (U/L)	549 ± 357	376 ± 168	0.27
KL-6 (U/mL)	1087 ± 584	1419 ± 1756	0.46
IgG (mg/dL)	1225 ± 398	1452 ± 454	0.27
Positivity for anti-CADM140/MDA5 Ab (%)	66.7	50.0	0.76
CT score (G)	2.1 ± 0.7	N/A	
CT score (F)	1.2 ± 0.6	N/A	

Data are mean ± SD or number of patients (percentage)

*DM* dermatomyositis, *RP-ILD* rapidly progressive-interstitial lung disease, *GC* glucocorticoid, *PaO*_*2*_*/FiO*_*2*_ partial arterial pressure of oxygen/fraction of inspired oxygen, *KL-6* Kerbs von Lungren 6 antigen, *CT* computed tomograpghy, *G* ground glass opacity, *F* fibrosis

The DM RP-ILD group included two patients positive for anti-PL-7 Ab (Additional file 1: Table S1), who developed RP-ILD during the course of remission maintenance therapy and thus, had long disease duration (35 and 120 months). One patient was treated with 7.5 mg/day PSL and the other with 3 mg/day TAC. The disease duration was long in the DM with RP-ILD group because this group not only included anti-MDA5 Abs-positive patients but also two anti-PL-7 antibody-positive patients.

### High serum IFN-γ, IL-1β, and IL-12 levels in patients with DM RP-ILD

**Fig. 2:**
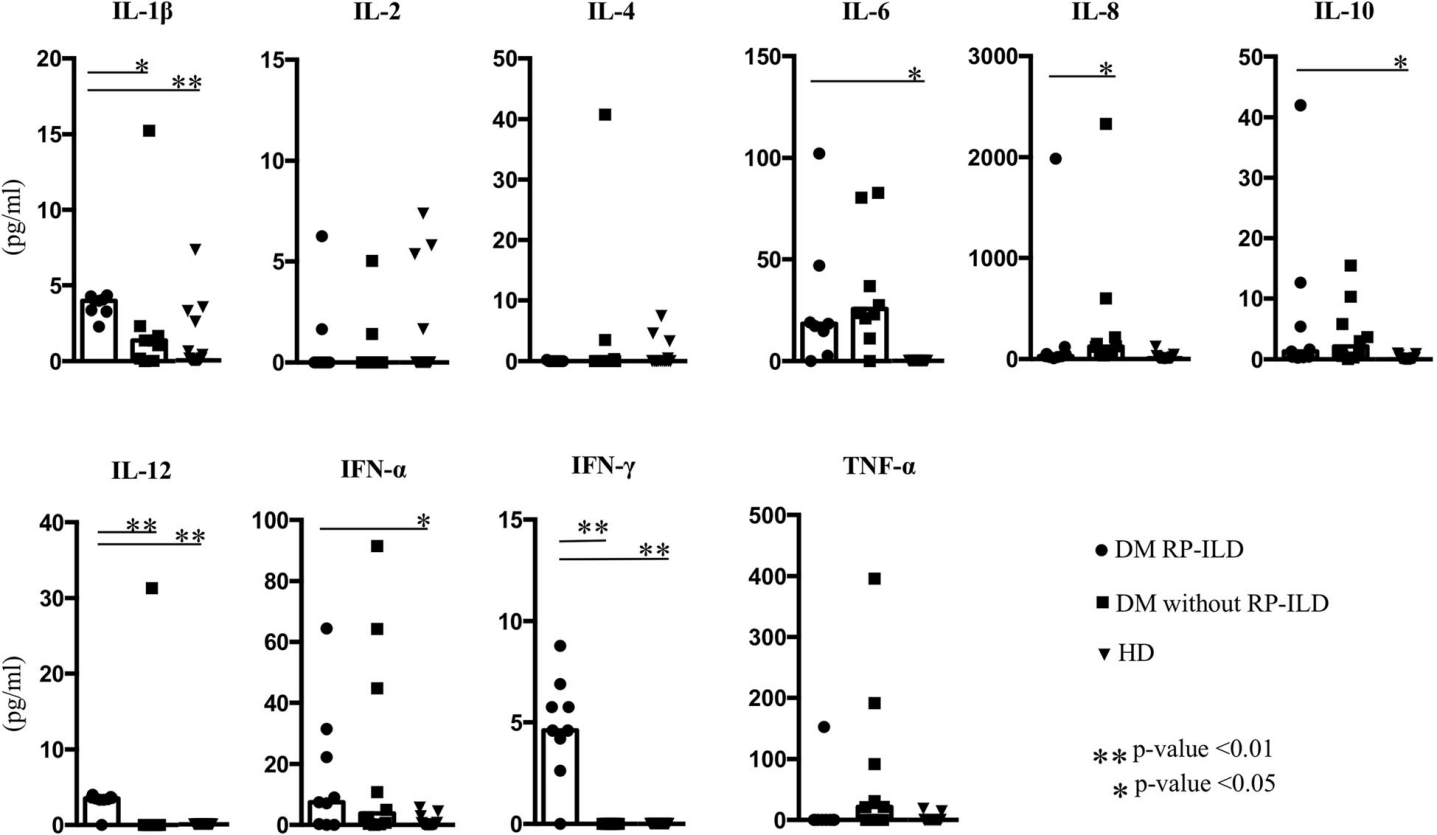
Serum levels of IL-1β, IL-2, IL-4 IL-6, IL-8, IL-10, IL-12, interferon (IFN)-α, IFN-γ and TNF-α in patients with dermatomyositis (DM), patients with DM complicated with rapidly progressive interstitial lung disease (DM RP-ILD) and healthy donors (HD). Symbols represent data of individual subjects. Statistical analysis is by Kruskal-Wallis followed by Dunn’s multiple comparison test

Figure 2 and Additional file 2: Table S2 compare serum cytokine levels among the DM RP-ILD, DM without RP-ILD, and HD groups, while Additional file 1: Table S1 shows antibody profiles and serum cytokine profiles in the same three groups. Serum levels of IFN-γ, IL-1β, and IL-12 were significantly higher in the DM RP-ILD group compared with the other two groups (IFN-γ, *p* < 0.01 vs DM without RP-ILD, and *p* < 0.01 vs healthy donors (HD); IL-1β, *p* = 0.03 vs DM without RP-ILD, and *p* < 0.01 vs HD; IL-12, *p* < 0.01 vs DM without RP-ILD and *p* < 0.01 vs HD). Furthermore, serum levels of IL-6, IL-10, and IFN-α were significantly higher in the DM RP-ILD group compared with the healthy donors, but were not significantly different from those in the DM without RP-ILD group. Interestingly, the serum levels of IL-2, IL-4, IL-8, and TNF-α levels were within the normal ranges in the DM RP-ILD group (Fig. 2). These results suggest that high serum levels of IFN-γ, IL-1β, and IL-12 are characteristic of DM RP-ILD. Unlike previous studies [17, 20, 21], our results showed no characteristic rises in IL-6, IL-8, IL-10, IFN-α, and TNF-α in DM RP-ILD.

### Serum IFN-γ levels correlate significantly with G-score in patients with DM RP-ILD

In addition to the high serum levels of IFN-γ (Fig. 2), in the DM RP-ILD group there was positive correlation between serum IFN-γ levels and the G-scores (ρ = 0.69, *p* = 0.04, Table 2). Although serum IL-1β also correlated significantly with the F-scores, the correlation was negative (ρ = − 0.68, *p* = 0.045, Table 2). None of the other cytokines were significantly correlated with the CT scores. These results demonstrate characteristically high serum IFN-γ in patients with DM RP-ILD, and significant correlation between IFN-γ and the G-score, which is a marker of the acute phase and disease activity in ILD. Moreover, the results suggest that IFN-γ plays a major role in the pathophysiology of DM RP-ILD.

**Table 2:** Association between CT scores and cytokines in patients with DM RP-ILD

	ρ	*p* value
CT scores (F)		
IFN-γ	0.10	0.80
IL-1β	− 0.68	0.05
IL-6	0.35	0.36
IL-12	− 0.14	0.71
TNF-α	− 0.43	0.25
IL-2	− 0.17	0.67
IL-4	− 0.07	0.85
IL-8	− 0.56	0.12
IL-10	−0.49	0.18
IFN-α	− 0.15	0.70
CT scores (G)		
IFN-γ	0.69	0.04
IL-1β	0.14	0.72
IL-6	0.24	0.53
IL-12	0.10	0.80
TNF-α	− 0.21	0.59
IL-2	− 0.45	0.22
IL-4	0.07	0.86
IL-8	− 0.12	0.76
IL-10	− 0.53	0.14
IFN-α	− 0.35	0.35

*DM* dermatomyositis, *RP-ILD* rapidly progressive-interstitial lung disease, *CT* computed tomography, *G* ground glass opacity, *F* fibrosis, *IFN* interferon, *IL* interleukin, *TNF* tumor necrosis factor

### Accumulation of IFN-γ-positive histiocytes in lungs and hilar lymph nodes, and IL-6-positive plasma cells in hilar lymph nodes in patients with DM RP-ILD

**Fig. 3:**
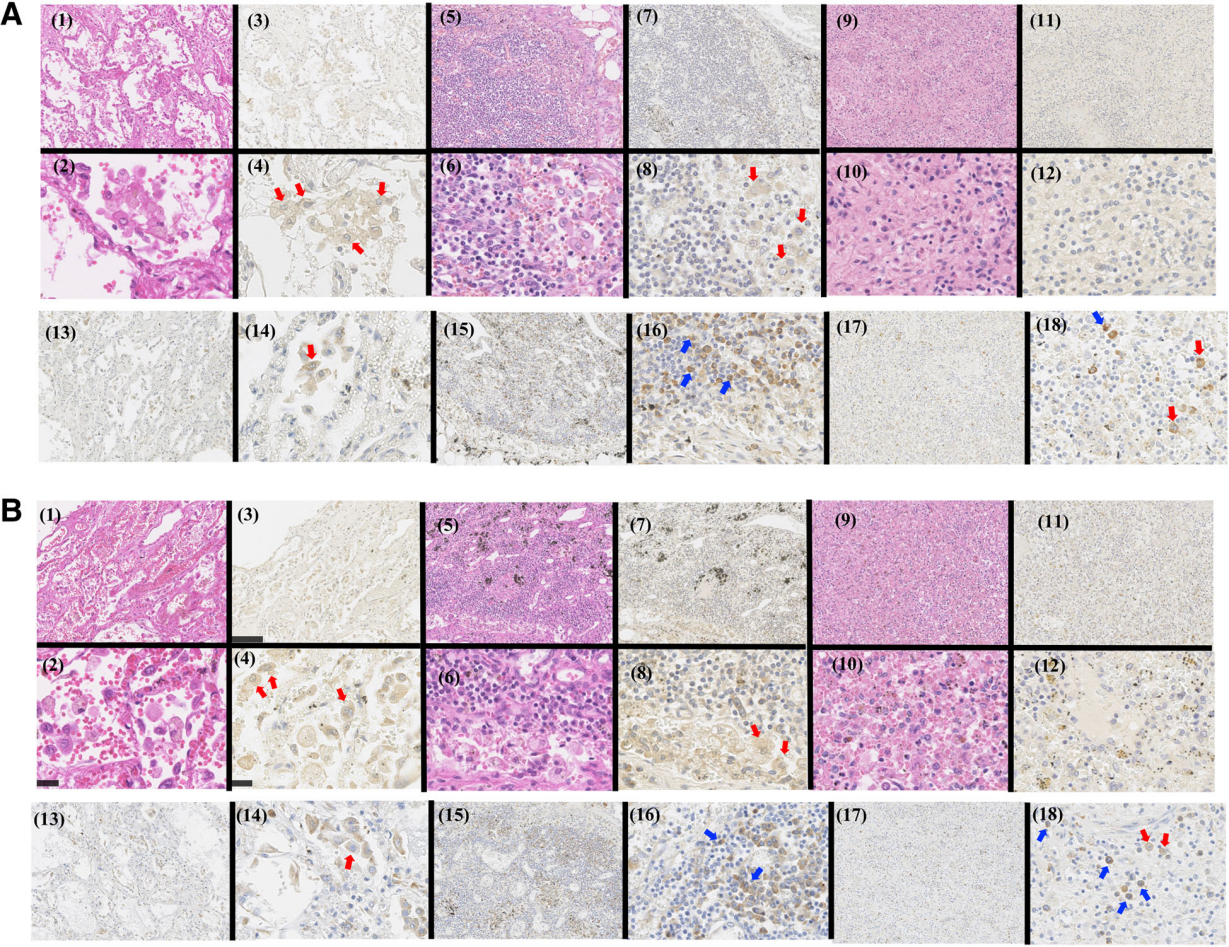
Histopathological findings from two autopsies. **a** Case 1: (1, 2) lung; hematoxylin-eosin (H&E) staining; (3, 4) lung, immunostaining for interferon (IFN)-γ; (5, 6) hilar lymph nodes, H&E staining; (7, 8) hilar lymph nodes, immunostaining for IFN-γ; (9, 10) spleen, H&E staining; (11, 12) spleen immunostaining for IFN-γ; (13, 14) lung, immunostaining for interleukin (IL)-6; (15, 16) hilar lymph nodes, immunostaining for IL-6; (17, 18) spleen, immunostaining for IL-6. **b** Case 2: (1, 2) lung, H&E staining; (3, 4) lung, immunostaining for IFN-γ; (5, 6) hilar lymph nodes, H&E staining; (7, 8) hilar lymph nodes, immunostaining for IFN-γ; (9, 10) spleen, H&E staining; (11, 12) spleen, immunostaining for IFN-γ; (13, 14) lung, immunostaining for IL-6; (15, 16) hilar lymph nodes, immunostaining for IL-6; (17, 18) spleen, immunostaining for IL-6. Red arrows show histiocytes, blue arrows show plasma cells

Serum IFN-γ was characteristically high and correlated with the G-scores in the DM RP-ILD group, whereas serum IL-6 was not elevated characteristically and did not correlate with the CT scores (Fig. 2 and Table 2), although IL-6 is reported to be important in DM RP-ILD [17, 19, 36, 37]. In the next step, we examined the roles of IFN-γ and IL-6 in the pulmonary pathophysiology of DM RP-ILD by immunostaining and hematoxylin-eosin (H&E) staining of lung tissues, hilar lymph nodes, and spleen tissues from two patients from whom specimens were obtained on autopsy (Fig. 3).

The first patient (Case 1) was a 70-year-old man with anti-MDA5 antibody-positive cADM. The patient was treated with four courses of GC pulse therapy, TAC, CsA, IVCY, and RTX (Fig. 3a). H&E staining showed diffuse hyaline membrane formation in the alveolar spaces. Fibroblast proliferation and incorporation of this hyaline membrane were observed in some parts, suggesting diffuse alveolar damage (DAD) extending from the exudative phase to the organizing phase (Fig. 3a (1)). Further analysis showed extravasation of erythrocytes and infiltration and aggregation of histiocytes in the alveolar spaces (Fig. 3a (2)). In these same tissues, IFN-γ-stained histiocytes had abundant cytoplasm and eccentrically distributed large nuclei (Fig. 3a (3, 4), red arrows). Marked infiltration of histiocytes was also noted into the lymph sinus of the hilar lymph nodes, with disappearance of nearly all lymphoid follicles (Fig. 3a (5, 6)). In addition to the lung tissue, IFN-γ-stained histiocytes were also found in the hilar lymph nodes (Fig. 3a (7, 8), histiocytes marked by red arrows). However, H&E staining of the spleen showed no marked histopathological changes other than splenic white pulp atrophy (Fig. 3a (9, 10)), with no infiltration of IFN-γ-positive histiocytes (Fig. 3a (11, 12)). The results of IL-6 staining are shown in Fig. 3a (13, 14, 15, 16, 17, 18). In the lung and spleen tissues, a few IL-6-positive histiocytes were observed, but cytoplasmic immunostaining was relatively weak (Fig. 3a (13, 14, 17, 18), histiocytes marked by the red arrow). Numerous IL-6-positive plasma cells were observed in hilar lymph nodes (Fig. 3a (15, 16), plasma cells marked by blue arrows). A small number of IL-6-positive histiocytes and plasma cells were also found in the spleen (Fig. 3a (17, 18), histiocytes and plasma cells are marked by the red and blue arrows).

The other autopsy specimen was from a 65-year-old woman with anti-PL-7 antibody-positive DM treated with one course of GC pulse therapy and IVCY (Fig. 3b). At the onset of RP-ILD, she was treated with 7.5 mg/day PSL, followed by a course of GC pulse therapy, TAC, and IVCY. The histopathological findings were similar to those of the first patient, despite different treatment histories and types of MSA. These results suggest that the pathophysiology of DM RP-ILD seems to be characterized by local appearance of IFN-γ-positive histiocytes in the lung tissues and related lymphoid tissues and the appearance of IL-6-positive plasma cells in hilar lymph nodes, regardless of the treatment history and type of MSA.

## Discussion

The present study demonstrated the presence of characteristically high serum IFN-γ in patients with life-threatening DM RP-ILD and that such levels correlated significantly with CT scores and histopathologic findings of pulmonary lesions. While high serum IL-6 reported in previous studies was also observed in patients with DM without RP-ILD, this finding might not be a characteristic of DM RP-ILD. Our results also showed significant correlation between serum IFN-γ levels and CT scores/G-scores, and infiltration of IFN-γ-positive histiocytes into the lung and hilar lymph node tissues, but not in the spleen, in patients with high disease activity. Numerous IL-6-positive plasma cells were also observed in the hilar lymph nodes but not in the lung. In the DM RP-ILD group, serum IFN-γ was elevated even in anti-MDA5 antibody-negative cases, whereas in the DM without RP-ILD group, serum IFN-γ was not elevated even in the majority of anti-MDA5 antibody-positive cases (Additional file 1: Table S1 and Additional file 2: Table S2). These results suggest that high serum IFN-γ is associated with the onset of RP-ILD, regardless of the presence of anti-MDA5 antibodies in patients with DM. Gono et al. [19] reported that anti-MDA5 antibody-positive ILD patients with high disease activity and poor prognosis tend to have a low IL-4/IFN-γ ratio, relative to patients with anti-ARS antibody-positive DM complicated with ILD. Considered together, these findings highlight the potential role of IFN-γ in the pathophysiology of anti-MDA5 antibody-positive DM.

No common pathophysiological processes between MAS and DM RP-ILD have previously been described. MAS is a secondary hemophagocytic syndrome (HPS) or hemophagocytic lymphohistiocytosis (i.e., autoimmune-associated HPS), in which various vital organs are damaged due to abnormal production of pro-inflammatory cytokines, such as IFN-γ [38]. It has been reported that hyperferritinemia, which reflects macrophage activation, is observed in 82% of patients with MAS [39, 40]. Moreover, cytopenia and liver dysfunction were often observed in patients with MAS [39, 41]. On the other hand, serum ferritin levels correlated with disease activity in patients with anti-MDA5 antibody-positive DM complicated with RP-ILD [6], who often have liver dysfunction and cytopenia [6, 42, 43]. We identified high serum IFN-γ and the presence of IFN-γ-positive histiocytes in the lung in patients with DM RP-ILD. These results suggest that in addition to its importance in MAS, IFN-γ seems to have a pathological influence in DM RP-ILD by activating macrophages and accelerating inflammation. This is the first study to report the characteristic presence of high serum IFN-γ in DM RP-ILD and that these levels correlate with the severity of pulmonary lesions assessed by CT scores/G-scores and histopathological examination.

In DM, serum IL-6 levels correlate with disease activity [44], and the use of tocilizumab (TCZ) is effective in patients with refractory DM [45]. However, there is no information on whether TCZ is effective against DM complicated with ILD or DM RP-ILD. Our study showed that (1) serum IL-6 was not specifically high only in DM RP-ILD but also in patients with DM without RP-ILD; (2) unlike IFN-γ, high serum IL-6 did not correlate with CT scores; and (3) numerous IL-6-positive plasma cells were found in hilar lymph nodes but not in the lungs. These results suggest that while IL-6 is important in the pathogenesis of DM RP-ILD, it is unlikely to be involved in local lung injury. Although serum IL-1β levels also correlated significantly with F-scores, the correlation was negative. Correlation between serum IL-1β levels and disease activity and pulmonary lesions was examined in previous studies, but no significant correlation was detected [17]. Our results also showed no significant correlation among other cytokines and CT scores in DM RP-ILD.

We expected to find systemic autoimmune inflammation, particularly in secondary lymphoid tissues, such as the spleen, in patients with DM RP-ILD. However, contrary to our expectation, the presence of IFN-γ-positive histiocytes was limited to local regions of the lungs and pulmonary hilar lymph nodes showing diffuse DAD. Neither infiltration of IFN-γ-positive histiocytes nor histopathological changes suggestive of inflammation were observed in the spleen. Inflammation limited to localized regions of the lungs may be a significant finding that could influence the selection of the drug administration route in the future.

The present study has certain limitations. First, the number of enrolled patients was relatively small because RP-ILD is an uncommon disease. Thus, our findings need to be confirmed in larger cohort studies. Second, it is possible that immunosuppressive therapy itself altered serum cytokine levels in the present study.

## Conclusions

IFN-γ was characteristically high in patients with DM RP-ILD after the onset of life-threatening RP-ILD. Furthermore, serum IFN-γ levels correlated with GGO, as evaluated by CT. Our results also suggested that inflammation might occur in localized regions of the lungs. Considered together, the results suggest the high potential of IFN-γ involvement in the pathophysiology of DM, specifically in the formation of pulmonary lesions seen in RP-ILD. Further prospective studies in large numbers of patients are needed.

## Additional files

Additional file 1: **Table S1** Serum levels of various cytokines, antibody profiles, and treatment at the time of registration. (DOCX 27 kb)

Additional file 2: **Table S2** Median values and ranges of the measured cytokines. (DOCX 16 kb)

## Abbreviations

Abs: Antibodies
ARDS: Acute respiratory distress syndrome
ARS: Aminoacyl-transfer ribonucleic acid synthetase
BALF: Bronchoalveolar lavage fluid
cADM: Clinically amyopathic dermatomyositis
CsA: Cyclosporine-A
CT: Computed tomography
DAD: Diffuse alveolar damage
DM RP-ILD: Dermatomyositis with rapidly progressive interstitial lung disease
DM: Dermatomyositis
ELISA: Enzyme-linked immunosorbent assay
FiO_2_: Fraction of inspired oxygen
F-score: Fibrosis score
GC: Glucocorticoid
GGO: Ground glass opacity
G-score: Ground glass opacity score
H&E: Hematoxylin-eosin
HD: Healthy donors
ICU: Intensive care unit
IFN-α: Interferon alpha
IFN-γ: Interferon gamma
IL: Interleukin
IL-1β: Interleukin-1beta
ILD: Interstitial lung disease
IVCY: Intravenous cyclophosphamide
MAS: Macrophage activation syndrome
MDA5: Melanoma differentiation-associated gene 5
MSA: Myositis-specific antibody
PaO_2_: Partial pressure of arterial oxygen
PCP: Pneumocystis pneumonia
PM: Polymyositis
PSL: Prednisolone
RNA: Ribonucleic acid
RP-ILD: Rapidly progressive interstitial lung disease
RTX: Rituximab
TAC: Tacrolimus
TNF-α: Tumor necrosis factor-alpha
