# Supplementary material for: Relevance of interferon-gamma in pathogenesis of life-threatening rapidly progressive interstitial lung disease in patients with dermatomyositis
Source: Arthritis Res Ther. 2018 Oct 26;20:240. doi: 10.1186/s13075-018-1737-2 (PMC6235206; doi:10.1186/s13075-018-1737-2)
Supplement: Supplementary file 1 — Table S1 Serum levels of various cytokines, antibody profiles, and treatment at the time of registration. (DOCX 27 kb) [file 13075_2018_1737_MOESM1_ESM.docx]

Table S1. Serum levels of various cytokines, antibody profiles, and treatment at the time of registration

|  | IFN-γ* | IL-1β* | IL-6* | IL-12* | TNF-α* | IL-2* | IL-4* | IL-8* | IL-10* | IFN-α* | Anti-CADM140/MDA5 Ab | Anti-PL-7  Ab | Anti-Jo-1 Ab | Other MSAs | Treatment |
| --- | --- | --- | --- | --- | --- | --- | --- | --- | --- | --- | --- | --- | --- | --- | --- |
| RP-ILD(+) | | |  |  |  |  |  |  |  |  |  |  |  |  |  |
| DM | 6.89 | 3.36 | 46.96 | 3.65 | 0.00 | 0.00 | 0.00 | 29.56 | 0.43 | 0.30 | Negative | **Positive** | Negative |  | PSL 7.5 mg |
| DM | 2.63 | 3492 | 14369 | 4.02 | 152 | 6.25 | 0.00 | 1986 | 42.0 | 22.3 | **Positive** | Negative | Negative |  | none |
| DM | 4.22 | 3.97 | 19.00 | 3.58 | 0.00 | 1.66 | 0.00 | 27.6 | 1.32 | 0.00 | Negative | **Positive** | Negative |  | PSL 5 mg, TAC 3 mg |
| DM | 4.61 | 3.28 | 102 | 3.34 | 0.00 | 0.00 | 0.00 | 21.4 | 0.29 | 7.42 | **Positive** | Negative | Negative |  | none |
| DM | 5.76 | 4.27 | 14.8 | 3.40 | 0.00 | 0.00 | 0.00 | 121 | 0.43 | 0.00 | Negative | Negative | Negative |  | none |
| DM | 8.78 | 4.35 | 18.5 | 3.34 | 0.00 | 0.00 | 0.00 | 20.6 | 12.6 | 31.5 | **Positive** | Negative | Negative |  | none |
| DM | 4.61 | 4.05 | 17.3 | 3.71 | 0.00 | 0.00 | 0.21 | 8.02 | 0.34 | 7.15 | **Positive** | Negative | Negative |  | none |
| DM | 5.76 | 3.97 | 0.00 | 3.52 | 0.00 | 0.00 | 0.00 | 50.8 | 5.43 | 64.5 | **Positive** | Negative | Negative |  | none |
| DM | 0.00 | 2.29 | 2.67 | 0.00 | 0.00 | 0.00 | 0.00 | 46.2 | 1.63 | 9.07 | **Positive** | Negative | Negative |  | none |
| RP-ILD(-) | | |  |  |  |  |  |  |  |  |  |  |  |  |  |
| DM | 0.00 | 0.00 | 0.00 | 0.00 | 0.00 | 0.00 | 0.35 | 35.1 | 0.56 | 2.58 | **Positive** | Negative | Negative |  | none |
| DM | 0.00 | 1.37 | 23.1 | 0.00 | 0.00 | 0.00 | 0.00 | 151 | 0.07 | 0.74 | Negative | Negative | **Positive** |  | PSL 15 mg, AZA 50 mg |
| DM | 0.00 | 0.17 | 23.7 | 0.00 | 21.6 | 0.00 | 0.00 | 41.8 | 3.01 | 44.8 | **Positive** | Negative | Negative |  | none |
| DM | 64.5 | 87.1 | 3471 | 0.00 | 396 | 0.00 | 0.00 | 2331 | 10.3 | 64.2 | **Positive** | Negative | Negative |  | none |
| DM | 0.00 | 1.06 | 36.8 | 0.00 | 31.0 | 0.00 | 0.00 | 217 | 5.86 | 0.00 | Negative | Negative | **Positive** |  | none |
| DM | 0.00 | 15.2 | 21.0 | 31.3 | 92.0 | 5.03 | 40.8 | 126 | 15.5 | 91.6 | **Positive** | Negative | Negative |  | none |
| DM | 0.00 | 1.37 | 27.4 | 0.00 | 21.1 | 0.00 | 0.00 | 598 | 1.24 | 0.17 | Negative | Negative | Negative | **anti-OJ Ab** | none |
| DM | 0.00 | 1.68 | 82.9 | 0.00 | 0.00 | 0.00 | 0.00 | 60.4 | 3.69 | 0.39 | Negative | Negative | **Positive** |  | none |
| DM | 0.00 | 2.32 | 11.2 | 0.00 | 0.00 | 1.42 | 3.51 | 53.1 | 0.99 | 4.97 | Negative | Negative | Negative |  | none |
| DM | 0.00 | 0.03 | 80.41 | 0.00 | 191 | 0.00 | 0.00 | 122 | 0.32 | 10. 8 | **Positive** | Negative | Negative |  | none |
| HD |  |  |  |  |  |  |  |  |  |  |  |  |  |  |  |
| HD1 | 0.00 | 0.00 | 0.00 | 0.00 | 0.00 | 0.00 | 0.00 | 8.16 | 0.00 | 0.00 |  |  |  |  |  |
| HD2 | 0.00 | 0.00 | 0.00 | 0.00 | 0.00 | 0.00 | 0.00 | 5.55 | 0.00 | 0.00 |  |  |  |  |  |
| HD3 | 0.00 | 0.00 | 0.00 | 0.00 | 0.00 | 1.66 | 0.00 | 6.59 | 0.14 | 0.70 |  |  |  |  |  |
| HD4 | 0.00 | 0.00 | 0.00 | 0.00 | 0.00 | 0.00 | 0.00 | 5.07 | 0.00 | 0.00 |  |  |  |  |  |
| HD5 | 0.00 | 0.14 | 0.00 | 0.00 | 0.00 | 0.00 | 0.00 | 7.49 | 0.00 | 0.17 |  |  |  |  |  |
| HD6 | 0.00 | 0.06 | 0.00 | 0.00 | 0.00 | 0.00 | 0.05 | 8.21 | 0.00 | 0.00 |  |  |  |  |  |
| HD7 | 0.00 | 0.00 | 0.00 | 0.00 | 0.00 | 0.00 | 0.05 | 43.19 | 0.89 | 0.59 |  |  |  |  |  |
| HD8 | 0.00 | 0.37 | 0.00 | 0.00 | 0.00 | 0.00 | 0.00 | 7.83 | 0.15 | 0.00 |  |  |  |  |  |
| HD9 | 0.00 | 3.51 | 0.00 | 0.00 | 18.58 | 7.38 | 7.44 | 11.76 | 0.70 | 5.71 |  |  |  |  |  |
| HD10 | 0.00 | 0.00 | 0.00 | 0.00 | 0.00 | 0.00 | 0.00 | 4.65 | 0.10 | 0.26 |  |  |  |  |  |
| HD11 | 0.00 | 7.30 | 0.00 | 0.00 | 14.19 | 5.37 | 4.60 | 9.83 | 0.00 | 4.48 |  |  |  |  |  |
| HD12 | 0.00 | 3.25 | 0.00 | 0.00 | 0.00 | 5.81 | 3.33 | 9.36 | 0.99 | 2.87 |  |  |  |  |  |
| HD13 | 0.00 | 0.60 | 0.00 | 0.00 | 0.00 | 0.00 | 0.00 | 9.26 | 0.10 | 0.00 |  |  |  |  |  |
| HD14 | 0.00 | 0.00 | 0.00 | 0.00 | 0.00 | 0.00 | 0.00 | 8.35 | 0.00 | 0.08 |  |  |  |  |  |
| HD15 | 0.00 | 0.00 | 0.00 | 0.00 | 0.00 | 0.00 | 0.00 | 16.77 | 0.12 | 0.26 |  |  |  |  |  |
| HD16 | 0.00 | 0.06 | 0.00 | 0.00 | 0.00 | 0.00 | 0.00 | 32.15 | 0.24 | 0.00 |  |  |  |  |  |
| HD17 | 0.00 | 0.00 | 0.00 | 0.00 | 0.00 | 0.00 | 0.49 | 11.42 | 0.20 | 0.13 |  |  |  |  |  |
| HD18 | 0.00 | 0.14 | 0.00 | 0.00 | 0.00 | 0.00 | 0.00 | 17.94 | 0.00 | 0.17 |  |  |  |  |  |
| HD19 | 0.00 | 2.56 | 0.00 | 0.00 | 0.00 | 0.00 | 0.00 | 122.65 | 0.17 | 0.00 |  |  |  |  |  |

*In pg/ml. DM, dermatomyositis; RP-ILD, rapidly progressive interstitial lung disease; HD, healthy donor; MSA, myositis-specific antibody; AZA, azathioprine; PSL, prednisolone; TAC, tacrolimus
