# Supplementary material for: Relevance of interferon-gamma in pathogenesis of life-threatening rapidly progressive interstitial lung disease in patients with dermatomyositis
Source: Arthritis Res Ther. 2018 Oct 26;20:240. doi: 10.1186/s13075-018-1737-2 (PMC6235206; doi:10.1186/s13075-018-1737-2)
Supplement: Supplementary file 2 — Table S2 Median values and ranges of the measured cytokines. (DOCX 16 kb) [file 13075_2018_1737_MOESM2_ESM.docx]

Table S2. Median values and ranges of the measured cytokines.

|  | DM with RP-ILD | DM without RP-ILD | Healthy Donors | *p* value |
| --- | --- | --- | --- | --- |
| IFN-γ | 4.61 (4.22-5.76) | 0.00 (0.00-0.00) | 0.00 (0.00-0.00) | <0.01 |
| TNF-α | 0.00 (0.00-0.00) | 21.4 (0.0-76.7) | 0.00 (0.00-0.00) | <0.01 |
| IL-1β | 3.97 (3.36-4.27) | 1.37 (0.39-2.16) | 0.06 (0.00-0.49) | <0.01 |
| IL-2 | 0.00 (0.00-0.00) | 0.00 (0.00-0.00) | 0.06 (0.00-0.49) | 0.96 |
| IL-4 | 0.00 (0.00-0.00) | 0.00 (0.00-0.27) | 0.00 (0.00-0.05) | 0.45 |
| IL-6 | 18.5 (14.8-47.0) | 25.6 (21.5-69.5) | 0.00 (0.00-0.00) | <0.01 |
| IL-8 | 29.6 (21.4-50.8) | 124 (54.9-201) | 9.26 (7.67-14.3) | <0.01 |
| IL-10 | 1.32 (0.43-5.4) | 2.13 (0.67-5.32) | 0.10 (0.00-0.19) | <0.01 |
| IL-12 | 3.52 (3.34-3.65) | 0.00 (0.00-0.00) | 0.00 (0.00-0.00) | <0.01 |
| IFN-α | 7.42 (0.30-22.3) | 3.77 (0.47-36.3) | 0.13 (0.00-0.42) | <0.01 |

DM, dermatomyositis; RP-ILD, rapidly progressive-interstitial lung disease

IFN, interferon; IL, interleukin; TNF, tumor necrosis factor
